# Supplementary material for: Unsupervised learning of pixel clustering in Mueller matrix images for mapping microstructural features in pathological tissues
Source: Commun Eng. 2023 Dec 8;2:88. doi: 10.1038/s44172-023-00136-6 (PMC10956012; doi:10.1038/s44172-023-00136-6)
Supplement: Supplementary file 2 — Supplementary Information [file 44172_2023_136_MOESM2_ESM.pdf]

Supplementary Material

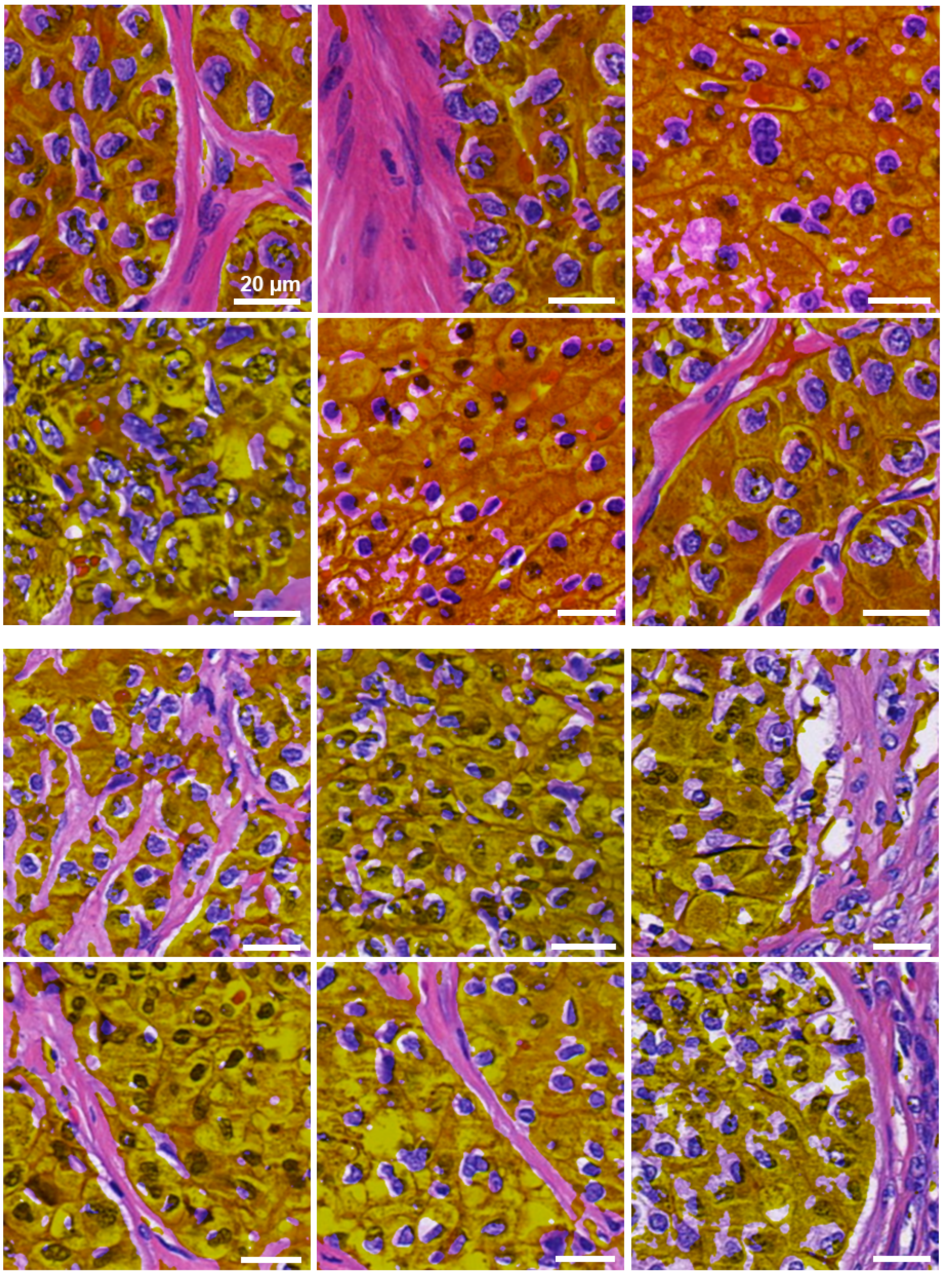

Supplementary Figure 1. Additional cluster 3 projection results on H&E images from both HCC and ICC ROIs, highlighted areas are the labelled areas of cluster 3, represented by a brown-red tint. Cluster 3 appears to be related to cytoplasm structures.

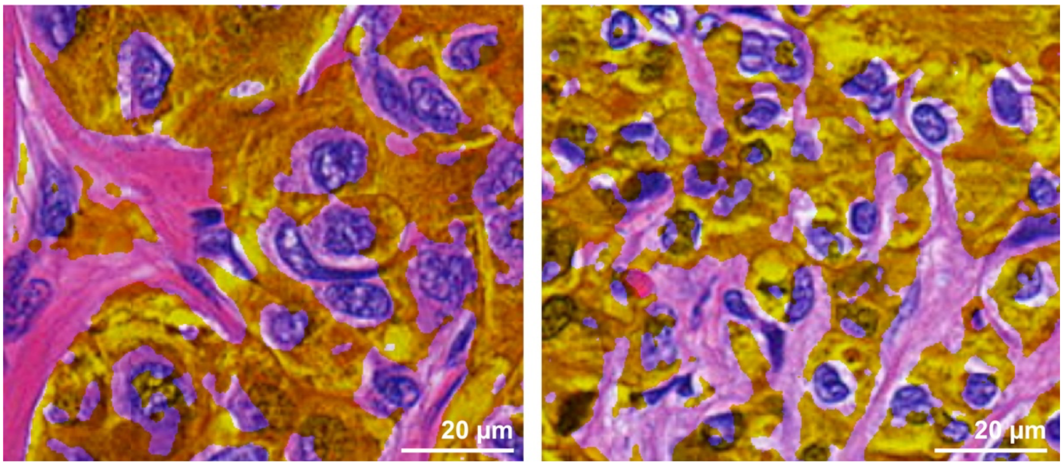

Supplementary Figure 2. Cluster 3 projection results on H&E images under 40X magnification, highlighted areas are the labelled areas of cluster 3, represented by a brown-red tint.

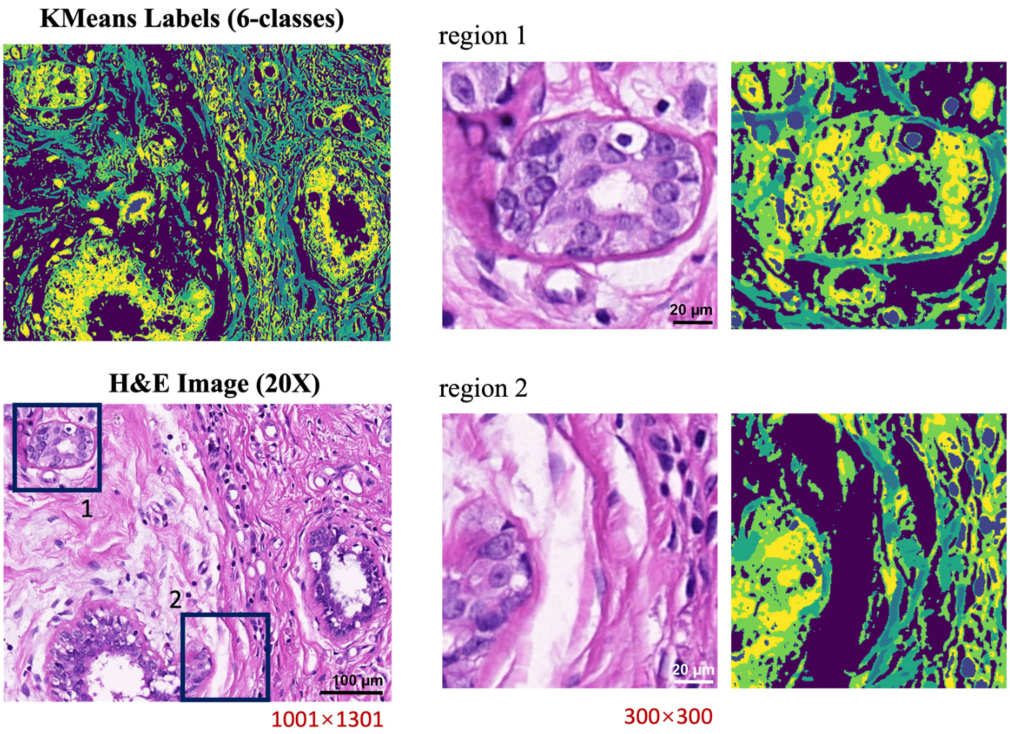

Supplementary Figure 3. KMeans clustering result on breast cancer specimen, with the KMeans cluster labels, corresponding H&E images, and two zoomed in regions for comparison.

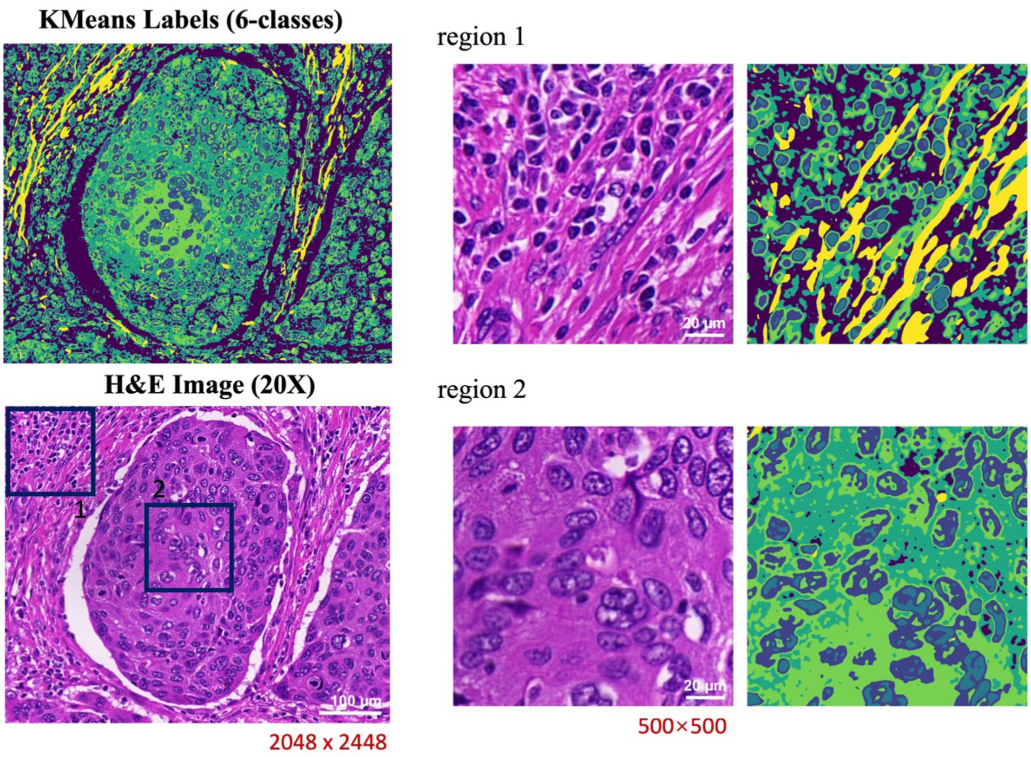

Supplementary Figure 4. KMeans clustering result on cervical cancer specimen, with the KMeans cluster labels, corresponding H&E images, and two zoomed in regions for comparison.

Polarization basis parameter	Description or explicit formula
B_b	$(M_{22} + M_{33})/2$
B_β	$(M_{23} - M_{32})/2$
$B_{\tilde{b}}$	$(M_{22} - M_{33})/2$
$B_{\tilde{\beta}}$	$(M_{23} + M_{32})/2$
t_1	$\sqrt{B_{\tilde{b}}^2 + B_{\tilde{\beta}}^2/2}$
$det\ B$	$M_{22}M_{33} - M_{23}M_{32}$
$norm\ B$	$\sqrt{(M_{22}^2 + M_{33}^2 + M_{23}^2 + M_{32}^2)}$
CD	$M_{14} + M_{41}$
P_L	$\sqrt{M_{21}^2 + M_{31}^2}$
D_L	$\sqrt{M_{12}^2 + M_{13}^2}$
r_L	$\sqrt{M_{24}^2 + M_{34}^2}$
q_L	$\sqrt{M_{42}^2 + M_{43}^2}$
$Transpose\ asym\ DP$	$\sqrt{(M_{12} - M_{21})^2 + (M_{13} - M_{31})^2}$
$Transpose\ asym\ rq$	$\sqrt{(M_{24} + M_{42})^2 + (M_{34} - M_{43})^2}$
$det\ MM$	Matrix determinant of Mueller matrix
$norm\ MM$	Matrix norm of Mueller matrix
$Trace\ MM$	Trace of Mueller matrix
$\lvert\vec{P}\rvert$	$\sqrt{M_{21}^2 + M_{31}^2 + M_{41}^2}$
$\lvert\vec{D}\rvert$	$\sqrt{M_{12}^2 + M_{13}^2 + M_{14}^2}$
$\vec{P} \cdot \vec{D}$	$M_{12}M_{21} + M_{13}M_{31} + M_{14}M_{41}$
$\vec{P}m\vec{D}$	Invariant under rotation and retarder transformation
$\vec{P}m^T\vec{D}$	Invariant under rotation and retarder transformation
λ_1	Eigenvalues of coherence matrix
λ_2	Eigenvalues of coherence matrix
λ_3	Eigenvalues of coherence matrix

λ_4	Eigenvalues of coherence matrix
P_1	Indices of polarimetric purity
P_2	Indices of polarimetric purity
P_3	Indices of polarimetric purity
PI	Overall purity index
PD	Depolarization index
S	Polarization entropy
Es	$M_{11} - M_{22} - M_{33} + M_{44}$
E_1	$(M_{11} + M_{22})^2 - (M_{12} + M_{22})^2 - (M_{33} + M_{44})^2 - (M_{34} - M_{43})^2$
E_2	$(M_{11} - M_{22})^2 - (M_{12} - M_{21})^2 - (M_{33} - M_{44})^2 - (M_{34} + M_{43})^2$
E_3	$(M_{11} + M_{21})^2 - (M_{12} + M_{22})^2 - (M_{13} + M_{23})^2 - (M_{14} + M_{24})^2$
E_4	$(M_{11} - M_{21})^2 - (M_{12} - M_{22})^2 - (M_{13} - M_{23})^2 - (M_{14} - M_{24})^2$
E_5	$(M_{11} + M_{12})^2 - (M_{21} + M_{22})^2 - (M_{31} + M_{32})^2 - (M_{41} + M_{42})^2$
E_6	$(M_{11} - M_{12})^2 - (M_{21} - M_{22})^2 - (M_{31} - M_{32})^2 - (M_{41} - M_{42})^2$
D	Diattenuation
Δ	Total depolarization
δ	Linear depolarization
R	Retardence
α	Circular dichroism

Supplementary Table 1. Full list and description of the polarization basis parameters. [1]

Supplementary References:

1. Li, P. *et al.* Polaromics: deriving polarization parameters from a Mueller matrix for quantitative characterization of biomedical specimen. *J. Phys. D: Appl. Phys.* **55**, 034002 (2021).
