# Supplementary material for: Unsupervised learning of pixel clustering in Mueller matrix images for mapping microstructural features in pathological tissues
Source: Commun Eng. 2023 Dec 8;2:88. doi: 10.1038/s44172-023-00136-6 (PMC10956012; doi:10.1038/s44172-023-00136-6)
Supplement: Supplementary file 5 — Reporting Summary [file 44172_2023_136_MOESM5_ESM.pdf]

nature portfolio

Corresponding author(s): Hui Ma

Last updated by author(s): Oct 31, 2023

Reporting Summary

Nature Portfolio wishes to improve the reproducibility of the work that we publish. This form provides structure for consistency and transparency in reporting. For further information on Nature Portfolio policies, see our Editorial Policies and the Editorial Policy Checklist.

Statistics

For all statistical analyses, confirm that the following items are present in the figure legend, table legend, main text, or Methods section.

n/a	Confirmed	
☐	☒	The exact sample size (*n*) for each experimental group/condition, given as a discrete number and unit of measurement
☐	☒	A statement on whether measurements were taken from distinct samples or whether the same sample was measured repeatedly
☐	☒	The statistical test(s) used AND whether they are one- or two-sided<br>*Only common tests should be described solely by name; describe more complex techniques in the Methods section.*
☐	☒	A description of all covariates tested
☐	☒	A description of any assumptions or corrections, such as tests of normality and adjustment for multiple comparisons
☐	☒	A full description of the statistical parameters including central tendency (e.g. means) or other basic estimates (e.g. regression coefficient) AND variation (e.g. standard deviation) or associated estimates of uncertainty (e.g. confidence intervals)
☒	☐	For null hypothesis testing, the test statistic (e.g. *F*, *t*, *r*) with confidence intervals, effect sizes, degrees of freedom and *P* value noted<br>*Give P values as exact values whenever suitable.*
☒	☐	For Bayesian analysis, information on the choice of priors and Markov chain Monte Carlo settings
☒	☐	For hierarchical and complex designs, identification of the appropriate level for tests and full reporting of outcomes
☒	☐	Estimates of effect sizes (e.g. Cohen's *d*, Pearson's *r*), indicating how they were calculated

Our web collection on statistics for biologists contains articles on many of the points above.

Software and code

Policy information about availability of computer code

Data collection	The data were collected using custom built Mueller matrix microscope using exsisting code pubslied in previous studies, as refereced in the main manuscript.
Data analysis	Data analysis is performed using Python 3.10. Specifically, the UMAP algorithm is implemented through the umap-learn package, and the minibatch KMeans algorithm is implemented through Scikit-learn package, version 1.2.0.

For manuscripts utilizing custom algorithms or software that are central to the research but not yet described in published literature, software must be made available to editors and reviewers. We strongly encourage code deposition in a community repository (e.g. GitHub). See the Nature Portfolio guidelines for submitting code & software for further information.

Data

Policy information about availability of data

All manuscripts must include a data availability statement. This statement should provide the following information, where applicable:

- Accession codes, unique identifiers, or web links for publicly available datasets
- A description of any restrictions on data availability
- For clinical datasets or third party data, please ensure that the statement adheres to our policy

The experimental data that support the findings of this study are available in Figshare with doi: dx.doi.org/10.6084/m9.figshare.23723490

Human research participants

Policy information about studies involving human research participants and Sex and Gender in Research.

Reporting on sex and gender	The findings of this study apply to both sex, since the biological attribute of sex was not a part of study design. Within the patients, 26 are male and 15 are female.
Population characteristics	The covariate-relevant polulation characteristics of the participents is based on their pathological diagnosis. The patients with HCC and ICC are selected, as well as their degree of cellular differentiation are recorded.
Recruitment	Pathologically diagnosed as HCC or ICC, using surgically removed tumour tissue.
Ethics oversight	The ethics committee of Fujian Cancer Hospital.

Note that full information on the approval of the study protocol must also be provided in the manuscript.

Field-specific reporting

Please select the one below that is the best fit for your research. If you are not sure, read the appropriate sections before making your selection.

☒ Life sciences ☐ Behavioural & social sciences ☐ Ecological, evolutionary & environmental sciences

For a reference copy of the document with all sections, see nature.com/documents/nr-reporting-summary-flat.pdf

Life sciences study design

All studies must disclose on these points even when the disclosure is negative.

Sample size	In this study 222 regions of interests were imaged using Muller matrix microscope, and used for analysis at pixel-level using unsupervised method. The selected sample size is reasonable for this study because the sample size is limited by algorithmic complexity, and the selected sample size is near the maximum allowed limit.
Data exclusions	Samples with unclear diagnosis, large area of necrosis, and defected pathological slide are not included. Patients with neo-adjuvant treatment are not included.
Replication	Study is repeated with increased sample size, and with different cancerous tissues such as cervical cancer and breast cancer tissue. In addition, for HCC and ICC samples we confirmed our observation with IHC staining.
Randomization	Continous surgical sample are selected, applied with mentioned recuitment criteria. All randomization are applied using Python random function.
Blinding	Blinding does not apply to out study.

Reporting for specific materials, systems and methods

We require information from authors about some types of materials, experimental systems and methods used in many studies. Here, indicate whether each material, system or method listed is relevant to your study. If you are not sure if a list item applies to your research, read the appropriate section before selecting a response.

Materials & experimental systems

n/a	Involved in the study	
☒	☐	Antibodies
☒	☐	Eukaryotic cell lines
☒	☐	Palaeontology and archaeology
☒	☐	Animals and other organisms
☒	☐	Clinical data
☒	☐	Dual use research of concern

Methods

n/a	Involved in the study	
☒	☐	ChIP-seq
☒	☐	Flow cytometry
☒	☐	MRI-based neuroimaging
